# Epidemiology of Psychiatric Risk in the Menopausal Transition: Subgroup Vulnerability and Population Incidence

**DOI:** 10.1192/j.eurpsy.2026.11950

**Published:** 2026-07-31

**Authors:** G. J. Cowen, A. Gasparova, N. Choudhury, B. R. Carr

**Affiliations:** 1 School of Medicine, Wayne State University, Detroit, MI; 2 Department of Psychiatry, University of Florida, Gainesville, FL, United States

## Abstract

**Introduction:**

The menopausal transition is a universal life stage for women, yet its psychiatric implications remain under-recognized in clinical practice. Epidemiological studies highlight perimenopause as a window of psychiatric vulnerability, yet both risk and protective factors remain insufficiently integrated into psychiatric training and clinical care.

**Objectives:**

To synthesize epidemiological evidence on risk and protective factors for psychiatric disorders in menopause, focusing on age at menopause, psychiatric history, and symptom severity.

**Methods:**

We conducted a narrative review of population-based and clinical studies (2005–2024) reporting psychiatric outcomes during perimenopause and postmenopause, including depression, anxiety, bipolar disorder, and sleep disturbance.

**Results:**

Women with symptomatic menopausal transitions show elevated risk for depression, anxiety, sleep disturbance, and bipolar disorder. Prior psychiatric history—especially major depressive disorder (MDD)—predicts recurrence, and combined MDD with childhood maltreatment confers heightened vulnerability in postmenopause. Prospective studies show recurrence risk for MDD during perimenopause is more than doubled in women with prior history (HR 2.67), but no consistent increase in first onset. Later menopause is associated with reduced depression risk, while severe vasomotor symptoms mediate risk across disorders. A large nationwide cohort (n=19,028) found symptomatic transition conferred significant risk for depressive (aHR 2.17), anxiety (aHR 2.11), bipolar (aHR 1.69), and sleep disorders (aHR 2.01), persisting beyond 5 years. Collectively, these findings position menopause as both a psychiatric risk amplifier and an opportunity for prevention.

**Conclusions:**

The menopausal transition can be a psychiatric risk state for vulnerable populations. Prospective studies show that recurrence of major depressive disorder is more than doubled in women with prior history (HR 2.67), particularly in the context of severe vasomotor symptoms and psychosocial stressors, while risk of first lifetime onset remains limited. Large-scale population data demonstrate that symptomatic menopausal transition independently predicts increased incidence of depressive (aHR 2.17), anxiety (aHR 2.11), sleep (aHR 2.01), and bipolar disorders (aHR 1.69), with effects persisting beyond five years. These findings suggest that menopause functions as a selective amplifier of psychiatric risk, underscoring the need for targeted screening, management of modifiable symptoms, and integration of menopause into psychiatric training and preventive care.

**Disclosure of Interest:**

None Declared

